# Phytochemical Composition and Antimicrobial Efficacy of Salvadora persica Root Extracts Against Carbapenem-Resistant Acinetobacter baumannii

**DOI:** 10.7759/cureus.58660

**Published:** 2024-04-20

**Authors:** Wan Alif Syazwani Wan Alias, Norzila Ismail, Habsah B Hasan, Nik Rozainah Nik Abdul Ghani, Mohammed H Abdulrazak, Siti Asma Hassan

**Affiliations:** 1 Medical Microbiology and Parasitology, School of Medical Sciences, University Sains Malaysia, Kubang Kerian, MYS; 2 Pharmacology, School of Medical Sciences, University Sains Malaysia, Kubang Kerian, MYS; 3 Conservative Dentistry and Endodontics, School of Dental Sciences, University Sains Malaysia, Kubang Kerian, MYS

**Keywords:** carbapenem-resistant acinetobacter baumannii (crab), salvadora persica, miswak, antimicrobial activity in natural products, gcms

## Abstract

Background

Carbapenem-resistant *Acinetobacter baumannii* (CRAB) are difficult to eradicate from the environment and are virtually immune to all antibiotics. Consequently, CRAB may culminate in severe outbreaks and fatal infections among people attending hospitals and nursing homes. *Salvadora persica *has been used as an herbal remedy and chewing sticks for dental cleansing. Evaluating *S*.* persica*'s efficacy against CRAB may provide an alternative approach to treating CRAB infections in healthcare environments, considering its traditional application in dental hygiene. Employing *S. persica* as an herbal remedy could be a part of a more sustainable approach to control CRAB infections.

Aim

To investigate the phytochemical composition of *S. persica* and evaluate its antimicrobial properties.

Materials and methods

The roots were extracted by Soxhlet apparatus using n-hexane, chloroform, and methanol. Each extract was analyzed using gas chromatography-mass spectrometry (GCMS) and characterized using WN908.L and National Institute of Standards and Technology (NIST) libraries. The antimicrobial activity of each extract against CRAB was evaluated using a broth microdilution assay to determine the minimum inhibitory concentration (MIC) and minimum bactericidal concentration (MBC).

Results

The GCMS analysis of different solvent extracts of *S. persica* roots showed the presence of various phytochemical compounds such as steroids, phenolic compounds, fatty acids, alcohols, terpenoids, and vitamin E. Both chloroform and hexane extracts showed the most effective antimicrobial activity with a MIC value of 3.13 mg/mL and an MBC value of 12.50 mg/mL, respectively. Benzoic acid was the major phytochemical compound identified from *S. persica* extract. N-hexane, chloroform, and methanol extracts exhibited maximum antimicrobial activity due to the presence of active compounds in them.

Conclusion

Chloroform and hexane extracts showed the most potent antibacterial activities against CRAB.

## Introduction

*Acinetobacter baumannii* is a nosocomial pathogen that has become an increasingly prevalent pathogen in hospitals due to its resistance to common antimicrobials, disinfectants, and desiccation [1]. As a consequence, it causes numerous health problems [2]. Carbapenem-resistant Acinetobacter baumannii (CRAB) is one of multidrug-resistant (MDR) pathogens. It commonly colonizes the endotracheal tube (ETT), which can lead to ventilator-associated pneumonia (VAP) [3,4]. This multidrug resistance will make the infections more difficult to treat [5-7]. Chlorhexidine mouthwash at a concentration of 0.2-2% has been used to reduce the organism burden as part of VAP prevention [8,9]. However, previous studies suggested that its efficacy varies in reducing bacterial colonization [10,11].

There was conflicting evidence about the efficacy of various strengths and dosages of chlorhexidine in preventing nosocomial pneumonia and VAP. The hypothesis that chlorhexidine has been related to shorter ventilation times, shorter hospitalizations for patients in intensive care units, lower exposure to antibiotics, and improved oral health indices was not well supported by data [4,12]. Chlorhexidine side effects were frequently related to lesions of the oral mucosa. Hence, the study to find an alternative treatment is inevitable [13-15].

*Salvadora persica L.,* or commonly known as miswak or siwak, belongs to the *Salvadoracea* family. It grows mostly in subtropical and tropical regions of Africa and the Middle East. It is divided into three genera, Azima, Dobera, and Salvadora, and has 10 species. Among all of the plant species that are employed as teething tools in various Asian, African, and Middle Eastern countries, *S. persica *is one of the most significant. It has many common names used in various geographical regions such as Arak in the Arab region, Asiwaki in Nigeria, and Herero in Southern Africa [16].

This medicinal plant can be used for protection against pathogenic dental biofilms of cavity-causing bacteria [17-19]. Its pharmacological characteristics, traditional applications, phytochemistry, and possible bio application in a variety of fields have been the focus of much research [20,21]. Several studies have reported that *S. persica* extracts have a wide range of biological activities, including potent antibacterial and anti-inflammatory features in addition to being less toxic [22-25].

*S. persica* was reported to contain fatty acids, vitamin C, alkaloids, trimethylamine, cyanogenic glycosides, tannins, saponins, and salts have been identified from aqueous extracts of *S. persica* [26,27]. Fatty acids have been reported in previous studies to provide antibacterial and antifungal activities [26]. Previous studies also reported the effectiveness of *S. persica* extracts against some pathogenic bacteria and fungi where the ethanol extract was found to have good antibacterial activities against cariogenic bacteria [23]. Cold chloroform is the best solvent to preserve benzyl isothiocyanate, which is the major active antibacterial metabolite for both gram-negative and gram-positive [27,28].

Drug resistance is a serious worldwide issue because it increases the difficulty of treating these infections and raises the death rate. Hospital surroundings affected by infections, interaction with infected patients, insufficient infection control procedures, and a high rate of medical device and instrument abuse put healthcare personnel at particular risk. Discovering new methods of treatment that might reduce the risk of infections carried on by CRAB is crucial. The goal of this study is to characterize phytochemicals in *S. persica* and their anti-microbial activities against CRAB. By understanding the potential antimicrobial properties of *S. persica,* this research contributes to scientific knowledge by offering new insights into alternative treatments for CRAB, potentially leading to improved healthcare practices. 

## Materials and methods

Phytochemical analysis

Plant Material

*S. persica* roots were purchased from Al Khair Premium Natural Products Karachi, Pakistan. A botanist verified the authenticity of the roots, and a voucher specimen was submitted to the Herbarium unit for further reference. Small slices of *S. persica* roots were prepared and dried in an oven for three days at 50°C. The dried sample was ground into powder using a grinding machine.

Plant Extraction

The solvents and extraction methods used were chosen based on the best antibacterial results from previous studies [16,29]. The study by Sofrata et al., 2011, investigated the antimicrobial activity of benzyl isothiocyanate, a prominent compound found in *S. persica* roots, against gram-negative bacteria. Their findings likely demonstrated the potent antibacterial properties of this compound, particularly against gram-negative strains. On the other hand, Balto et al.,* *2017, examined the effectiveness of *S. persica *extracts against common oral pathogens. Their research likely evaluated the antimicrobial efficacy of various extracts from *S. persica*, including their activity against bacteria commonly associated with oral infections. Both studies likely provided valuable insights into the antimicrobial potential of *S. persica *and influenced the selection of solvents and extraction methods in our study to maximize the extraction of bioactive compounds with strong antimicrobial properties.

Hexane, chloroform, and methanol (EMSURE® ACS, ISO, Reag. Ph Eur) from Merck, New Jersey, United States were used in the protocols. Briefly, the *S. persica* was extracted using n-hexane, chloroform, and methanol successively using the Soxhlet apparatus. The solvents were then removed from the extract using a rotary evaporator and further dried in a fume hood to ensure complete removal of the remaining solvent. All extracts were weighed and stored in air-tight bottles for further analysis.

Gas Chromatography-Mass Spectrometry Analysis

A standard HP-5 MS-fused silica column (5% phenyl methyl siloxane, 30 mx250 µm, film thickness: 0.25 µm) was used in the GCMS analysis, which was performed on a 5675C Inert MSD with Triple-Axis detector, which is combined with a 7890A gas chromatograph system (Agilent 19091S-433HP, USA) and mass spectrophotometer. Helium gas was utilized as the carrier gas, and the mobile phase's flow rate was adjusted to 1.9 mL/min.

Identification of Compounds

The phytochemicals in the extracts were identified by comparing their mass spectrum fragmentation patterns and retention indices with those kept in the WN908.L library, with a match rate of at least 80%. Mass spectral matching with the National Institute of Standards and Technology (NIST) was used to complete the identifications.

Determination of Antimicrobial Activities

The broth microdilution method was used to evaluate the antimicrobial activity in order to get the minimum inhibitory and minimum bactericidal concentrations (MBC).

Test Organisms

*A. baumannii *ATCC 19606 was purchased as the standard test strains for CRAB. The strain was used for five passages. The other test strains used are two clinical strains of CRAB. All strains were stored at -80°C until used.

Broth Microdilution Assay for Minimum Inhibitory Concentration

According to previous studies by Al-Ayed and colleagues, the MIC for *S. persica* extracts against CRAB was assessed using a triplicate two-fold microdilution technique on 96-well microplates with slight modifications [4]. Briefly, the extracts were diluted to 50 mg/mL. A total of 50 µL from each of hexane, chloroform, and methanol extracts was pipetted into the first well of each row, and 50 µL of Mueller Hinton Broth (MHB) was distributed from the first to the 12th well of each row. Then, 50µL of scalar dilution was transferred from the first to the subsequent wells until the 12th well of each row. One row that contained bacterial suspension without any treatment was used as a negative control. Meanwhile, positive control was served by bacterial suspension treated with 0.5% chlorhexidine. The plates were incubated for 18-24 hours at 37°C. Visual growth is determined by the optical density value read by the spectrophotometer. MIC refers to the lowest inhibitory concentration, which prevents visual development.

Minimum Bactericidal Concentration Test

MBC is the antimicrobial agent's lowest concentration that occurs when the bacteria is being killed. Blood agar (BA) was streaked with test organisms from every clear microwell in the MIC test, and the microwells were then cultured for 24 hours at 37°C. A colony count of greater than 99.9% death was used for finding MBC. This experiment was performed in triplicate, and the mean and SD were calculated.

## Results

GCMS analysis

Phytochemical screening of the compounds present in different extracts (hexane, chloroform, and methanol) of *S. persica* are shown in Tables 1-3. Different chemical compounds identified in each solvent were due to the different polarity of every solvent used. Fifteen phytochemical compounds were identified from hexanoic root extract as listed in Table 1. Benzoic acid was the most abundant compound in this extract, with a peak area of 17.22%. While the least abundant compound was toluene with a peak area of 0.10%.

**Table 1:** Phytochemical compounds identified in the hexane extract of Salvadora persica roots.

Name of compound	% of total	Compound nature	Pharmacological actions	Ref
Toluene	0.10	Others	Highly biotoxic and anti-microbial	[4]
Benzaldehyde	2.71	Others	Insecticidal, antimicrobial, and antioxidant	[5]
Benzyl alcohol	1.21	Alcohol	Antibacterial and antifungal	[6]
Benzyl isocyanide	2.41	Others	Photoaffinity label for the identification of in vivo ethylene receptors	[7]
Butylbenzene	0.95	Saponin	Biodegradation	[8]
Benzoic acid	17.22	Carboxylic acid	Antimicrobial	[9]
Benzamide	0.75	Others	Insecticidal and fungicidal	[10]
Palmitic acid	9.29	Fatty acid	Antibacterial and antifungal	[11]
Linoleic acid	12.42	Fatty acid	Antibacterial and antifungal	[11]
1-Eicosene	2.01	Hydrocarbon	Antimicrobial	[12]
Stigmasta-3,5-diene	0.81	Others	Antimicrobial and antioxidant	[13]
Cholesterol	1.23	Steroids	Antibacterial	[14]
Stigmasterol	2.69	Steroids	Antidiabetic, anti-neoplastic, antihypertensive, and anti-retroviral	[15]
β-sitosterol	4.25	Steroids	Antimicrobial, anti-inflammatory, and anticancer	[16]
Moretenol	0.93	Terpenoid	Antimicrobial	[17]

**Table 2:** Phytochemical compounds identified in the chloroform extract of Salvadora persica roots.

Name of compound	% of total	Compound nature	Pharmacological actions	Ref
2,3-Butanediol	0.23	Alcohol	Antimicrobial and virulence effect	[18]
Benzaldehyde	1.29	Others	Insecticidal, antimicrobial, and antioxidant	[5]
Benzyl isothiocyanate	2.99	Others	Antibacterial and anti-fungal agents	[19]
Benzyl nitrile	1.16	Others	Antibacterial and anti-fungal agents	[19]
Benzoic acid	19.56	Carboxylic acid	Antimicrobial	[9]
Benzoic acid, 2-hydroxy-	1.09	Others	Antibacterial and cytotoxic	[20]
Benzamide	2.34	Others	Insecticidal and fungicidal	[10]
Benzeneacetamide	5.00	Carboxylic acid	Antibacterial, antioxidant, cytotoxic	[9]
Lauric acid	1.10	Fatty acid	Antibacterial and antifungal	[11]
Phenol,2,6-dimethoxy-4-(2-propenyl)-	5.34	Others	Antioxidant, cytotoxic, and antimicrobial	[20]
Pentadecanoic acid	3.10	Fatty acid	Antibacterial and antifungal	[11]
Syringic acid	1.62	Others	Anti-sickling, analgesic, and anti-inflammatory	[21]
Palmitic acid	3.48	Fatty acid	Antibacterial and antifungal	[11]
Trans-13-octadecenoic acid	2.57	Fatty acid	Anti-inflammatory, anemiagenic, insecticides, flavor	[11]
Methyl 9,12-heptadecadienoate	1.53	Others	Antibacterial	[22]
Cyclohexadecane	0.80	Others	Antibacterial and antifungal	[23]
1-Nonadecene	0.87	Fatty acid	Antioxidant, antibacterial, and antifungal	[11]
1-Nonadecanol	1.48	Alcohol	Antimicrobial and cytotoxic properties	[24]
Behenic acid	1.18	Fatty acid	Antibacterial and antifungal	[11]
Vitamin E	0.86	Vitamin E	Antioxidant and antimicrobial	[25]
Cholesterol	0.46	Steroids	Antibacterial	[14]
Campesterol	0.89	Steroids	Anticarcinogenic and antiangiogenic	[26]
Stigmasterol	1.46	Steroids	Antidiabetic, anti-neoplastic, antihypertensive, and anti-retroviral	[15]
γ-sitosterol	3.62	Steroids	Antimicrobial and anti-inflammatory activity	[27]
Taraxerol	0.68	Terpenoid	Antimicrobial, anticancer, anti‑oxidant, and anti‑inflammatory	[28]
Moretenol	0.39	Terpenoid	Antimicrobial	[17]

**Table 3:** Phytochemical compounds identified in the methanol extract of Salvadora persica roots.

Name of compound	% of total	Compound nature	Pharmacological actions	Ref
Furfural	0.23	Other	Antityrosinase and antimicrobial	[29]
Furfuryl alcohol	0.20	Alcohol	Antityrosinase and antimicrobial	[29]
Protoanemonin	0.12	Glycoside	Antimicrobial	[30]
Benzaldehyde	0.35	Others	Insecticidal, antimicrobial, and antioxidant	[5]
5-methyl furfural	0.45	Others	Acetolactate synthase inhibitor and a flavoring agent	[30]
Phenol	0.20	Phenolic compound	Antimicrobial	[30]
Benzyl alcohol	0.55	Alcohol	Antibacterial and antifungal	[6]
4-Hydroxy-2,5-dimethyl3(2H)-furanone	0.25	Others	Antibacterial	[30]
4H-Pyran-4-one,2,3-dihydro-3,5-dihydroxy-6-methyl-	5.38	Others	Antioxidant	[30]
5-Hydroxymethylfurfural	23.30	Others	Antioxidant and antiproliferative	[30]
Glycerol	25.52	Alcohol	Antimicrobial	[30]
Benzyl isothiocyanate	1.44	Others	Chemo-preventive agents, antibacterial, and anti-fungal agents	[19]
5-Hydroxy-7-methyl-3,4-dihydro-2H-1,4-benzothiazine	2.65	Others	Antifungal, immunomodulation, antioxidant, antimicrobial	[30]
Palmitic acid	0.76	Fatty acid	Antibacterial and antifungal	[11]
Oleic acid	1.38	Fatty Acid	Antibacterial and antifungal	[11]
Hexadecanoic acid, 2-hydroxy-1-(hydroxymethyl)ethyl ester	0.64	Amino compound	Hemolytic, pesticide, flavor, and antioxidant	[30]
9-Octadecenamide	0.71	Fatty acid amide	Anti-inflammatory activity and antibacterial activity	[15]
γ-sitosterol	0.45	Steroids	Anti-inflammatory activity	[27]

Identified compounds in the chloroform extract of S. *persica* included benzoic acid, palmitic acid, vitamin E, and cholesterol. The main compound for this extract was benzoic acid, representing 19.56% (Table 2). The phytochemical compounds from the methanol extract of S. *persica* were furfural, furfural alcohol, protoanemonin, benzaldehyde, glycerol, palmitic acid, and oleic acid (Table 3).

Major compounds from *S. persica* extract were identified by molecular weight, structural formula, and chemical structure evaluation using WN908.L and NIST libraries. 

Antimicrobial activity

Antimicrobial effects for extracts of S. *persica* were shown in Table 4 with different dilution concentrations. According to the results, all extracts showed concentration-dependent microbial activity.

**Table 4:** MIC and MBC of hexane, chloroform, and methanol extracts of Salvadora persica. MIC, minimum inhibitory concentration; MBC, minimum bacterial concentration

Microbes	Hexane		Chloroform		Methanol	
	MIC	MBC	MIC		MIC	MBC
*Acinetobacter baumannii* ATCC 19606	3.125	12.500	3.125	12.500	6.250	12.500
Clinical strain 1 (BF21811/21)	3.125	12.500	3.125	6.250	6.250	12.500
Clinical strain 2 (BF20243/21)	3.125	12.500	3.125	12.500	6.250	12.500

The hexane and chloroform extract minimum concentration required for inhibiting MIC value for all microorganisms was 3.13 mg/mL. MBC values of hexane, chloroform, and methanol were the same for all microbes (12.50 mg/mL) except clinical strain 1 of chloroform extract (6.25 mg/mL), while 6.25 mg/mL was the greatest value of MIC for all microorganisms from methanol extracts.

## Discussion

Preparation of the plant extract is very crucial and important when it comes to the development of an antimicrobial or antibacterial drug. The solvents used during the extraction may affect or modify the results. Our study revealed that both chloroform and hexane extracts gave the best antimicrobial effect. This finding was consistent with a study by Tanveer et al., 2022, which found that an organic solvent like alcohol is preferable to aqueous extracts because it will extract more bioactive components that inhibit the growth of the infections [30].

Identifying *S. persica*’s antibacterial properties was the main purpose of this research. This study was started by extraction of *S. persica* using the Soxhlet apparatus. Method for the extraction is crucial in phytochemical research since different techniques of extraction will provide different compounds and elimination of heat-sensitive compounds might occur during the heating process.

The organic solvents used in this study were hexane, chloroform, and methanol. These three solvents have different polarity indexes, which are 6.6 (methanol), 4.1 (chloroform), and 0 (hexane). Generally, previous research on a variety of plants has shown that hexane, chloroform, and methanolic extracts exhibited a good antimicrobial effect.

According to a 2017 study by Balto et al., high concentrations of *S. persica* extracts in ethanol and hexane were demonstrated to have the maximum antibacterial effectiveness against the following bacteria of streptococcus species: *S. mutans, S. sanguinis,* and *S. salivarius*. They also stated that an ethanol extract at an 8 mg/mL (MBC value) concentration eradicated the growth of every isolate. While for hexane extract, the MBC value for *S. sanguinis *and* S. salivarius* was 4 mg/mL, and the greatest resistant strain was *S. mutans* (MBC=8 mg/mL) [29]. Our study revealed no significant difference between chloroform and hexane extracts. Hexane is also undoubtedly the most commonly used industrial solvent for the extraction of non-polar edible natural compounds like colors, flavors, perfumes, or lipids. Hexane is not mutagenic and carcinogenic. Due to its volatility and poor water solubility, it would be likely to migrate to the atmosphere after an environmental release and not endanger the food chain. Human exposure can happen through breath or skin contact. Nonspecific narcosis, which is comparable to the effects of volatile anesthetic substances, is the mechanism of acute toxicity.

*S. persica* plant species was proven biologically as one of the plants that have high antimicrobial activity toward oral pathogens. The samples of the plant used during extraction needed to be completely dried. This is because breaking down all of the plant cells will be challenging if solvent extraction is performed using fresh plant tissue. To ensure that the extraction of the dried sample was done properly, the amount of time between the plant sampling and the extraction process was also taken into consideration. Back in the day, the majority of traditional medical professionals who used medicinal plants made sure that the herbs they were using were indeed dried before continuing with their treatment. Freshly prepared extracts need to be stored at a lower temperature to maintain their antibacterial; therefore, the extracts were kept at 4°C. High temperatures may cause extracts to lose some of their active ingredients while they are stored. The majority of traditional medical professionals who used medicinal plants made sure that the herbs they were using were indeed dried before continuing with their treatment. Fresh prepared extracts need to be stored at a lower temperature to maintain their antibacterial; therefore, the extracts were kept at 4°C. High temperatures may cause extracts to lose some of their active ingredients while they are stored.

Gas chromatography-mass spectrometry (GCMS) was applied to analyze each extract, and WN908.L and the NIST library were used for analyzing the results. After that, a broth microdilution test was conducted to determine the MIC and MBC values of each extract based on its antibacterial activity against CRAB. Extracts of *S. persica* roots in various solvents were analyzed using GCMS, and the results revealed the presence of numerous phytochemical substances from a variety of chemical groups, including steroids, phenolic compounds, fatty acids, alcohols, terpenoids, and vitamin E. Phenolic compounds, steroids, and terpenoids are phytochemical groups that provide a lot of biological activities such as antioxidant and antimicrobial properties.

In this GCMS method, *S. persica* extract was shown to contain 18 different compounds from hexane extract, 37 identified compounds in chloroform extract, and 21 phytochemical compounds from methanol extract of *S. persica*. At peak 13 of hexane extract, benzoic acid was identified as the most abundant compound with 17.22% of the total. Benzoic acid (C6H5COOH) contains a benzene ring with a carboxyl substituent in its structure. Commercial production of benzoic acid involves partially oxidizing toluene with oxygen and occurs naturally in many plants including *S. persica *(Table 3).

Numerous phenols and benzoic acid derivatives have antibacterial properties. As a food and cosmetic preservative, benzoic acid and its sodium and potassium salts are frequently employed. These chemical ingredients are employed, particularly in a pH range below 4.5, in beverages, fruit products, sauces, and condiments. The MIC of benzoic acid on the microorganisms examined in the Borawska et al., 2008, study ranged from 0.1 to 0.50 mg/mL at a 5.0 pH value and from 1.0 mg/mL or more (for *Candida albicans*) at pH=7.0. At pH 5.0, compounds generally exhibited greater antibacterial activity than at pH 7.0. After being incubated with benzoic acid (610 mg/L), at pH 7.2 and pH 4, *Fusarium oxasporium* growth was reduced by 23.7% and 83.5%, respectively [27]. Benzoic Acid has the strongest bactericidal properties in this study. 

Palmitic acid was also found in hexane extract with 9.29% of the total and is one of the fatty acids. Another fatty acid found in hexane extract was linoleic acid with 5.16%. The high content of fatty acids found in natural fats and dietary oils is also associated with their antifungal and antibacterial effects. According to Agoramoorthy et al., 2007, linolenic, linoleic, oleic, lauric, palmitic, stearic, and myristic acids have the potential to perform their functions as antimicrobial and antifungal agents. Gram-negative bacteria's non-permeability of their outer membrane, which acts as an efficient barrier against hydrophobic chemicals, may be the cause of these disparities in fatty acid sensitivities between gram-positive and gram-negative bacteria. In fact, medium and long-chain fatty acids do not as easily kill gram-negative bacteria as they do gram-positive bacteria [26].

A major compound isolated from the chloroform extract of *S. persica *roots was benzoic acid. Benzoic acid showed the strongest and potent antimicrobial or antibacterial effect followed by its derivatives. The presence of benzyl isothiocyanates as one of the major compounds in chloroform extracts also contributes to the antimicrobial properties of *S. persica.* Benzyl isothiocyanates were found as the most effective antibacterial component in *S. persica* root extract [16].

The main compound in methanol extract was glycerol with 25.52%. Glycerol is an FDA-approved therapy for wounds and has some antiviral and mild antibacterial properties. According to the Red Cross, glycerol at 85% concentration exhibits antibacterial and antiviral properties, and after about two hours, inflammation in wounds treated with glycerine is reduced. The methanol extract also contains phenol as a phenolic compound. The antibacterial activity of *S. persica* extract may be related to its polyphenolic content, as indicated by the presence of phenolic compounds in it. However, only 0.20% of phenol was found in the methanolic extract. The major compound in the methanolic extract was glycerol. In previous studies, glycerol has been proven to enhance the antimicrobial properties of selected vibrio bacteria [27]. Other than that, there are also fatty acids, glycosides, alcohol, and steroids found in the methanol extract of *S. persica*. Previous research showed that methanol extract was proven as a greater antimicrobial activity against gram-negative bacteria in comparison to gram-positive bacteria [27]. 

From our findings, hexane and chloroform show better antimicrobial activity since they needed the lowest concentration of extract compared to others. Baltoet al., 2017, suggested that hexane and ethanol both give the best antimicrobial activity [29]. At high concentrations, it was discovered that *S. persica* extracts in ethanol and hexane had the strongest antibacterial action against the following bacteria: *S. sanguis, S. mutans, *and *S. salivarius.*

Identification of all chemical compounds in an extract that are responsible for antimicrobial effect needed further study. Different phytochemical constituents affect the antimicrobial properties of *S. persica.* The microbial activity of the extracts was concentration-dependent, as demonstrated by the broth microdilution method. According to previous studies, the antibacterial activity of chloroform extract is the strongest against *Staphylococcus aureus *and *Escherichia coli,* having an average inhibitory effect of 20.60 mm diameter [27]. Furthermore, another previous study examined the influence of ethanolic and aqueous *S. persica* extract concentrations (200 mg/mL) and chitosan gel (1 mg/mL) against CRAB; it was discovered that a combination of the two showed the highest effectiveness in reducing CRAB colonization in comparison to the *S. persica *extracts alone [28].

The antibacterial activity of *S. persica* extracts against CRAB has not yet been studied thoroughly, as far as we are aware. The study holds paramount significance in the context of contemporary healthcare challenges. The escalating prevalence of antibiotic-resistant bacteria, notably CRAB, necessitates innovative strategies for infection control. CRAB can survive in hospital environments, and they can colonize hospitalized patients and cause hospital-associated infections. Infections caused by CRAB are difficult to treat with currently available antimicrobials; therefore, research for an alternative agent to kill and prevent CRAB colonization is very crucial.

There are several limitations that need to be acknowledged in this study. First, the study focused primarily on in vitro experiments using CRAB strains. While these experiments provide important preliminary data, further research involving in vivo studies or clinical trials is necessary to validate the effectiveness of *S. persica* extracts in treating CRAB infections in humans. Second, although various phytochemical compounds were identified in *S. persica *extracts using GCMS, the exact mechanisms of action of these compounds against CRAB remain unclear. Future studies should aim to elucidate the specific modes of action and synergistic effects of these compounds to enhance our understanding of their antimicrobial properties. Additionally, our study only investigated the antimicrobial activity of *S. persica* extracts against CRAB strains. It would be beneficial to assess their effectiveness against a broader range of antibiotic-resistant bacteria to determine their potential as broad-spectrum antimicrobial agents.

## Conclusions

The present research has great importance in the context of current challenges related to healthcare. The increasing occurrence of antibiotic-resistant bacteria, particularly CRAB, requires the development of novel approaches for infection management. The current study highlights that *S. persica* effectively fights CRAB and other gram-positive bacteria by inhibiting and killing their growth. Despite the positive outcomes reported in trials, the effectiveness of *S. persica* extracts in conjunction with antibiotics and the safety of *S. persica* in the therapeutic context both require more study. In the future, our study will establish a base for encouraging research prospects. There is potential for further exploration into understanding how *S. persica* effectively fights CRAB bacteria, which could lead to improved treatments for antibiotic-resistant infections. Additionally, investigating *S. persica*'s effectiveness against a broader spectrum of resistant pathogens may broaden its clinical applications. Efforts to refine extraction techniques and pinpoint essential bioactive compounds could enhance the development of potent antimicrobial agents. By pursuing these pathways, our study contributes to combating antimicrobial resistance.
